# On/off-switchable LSPR nano-immunoassay for troponin-T

**DOI:** 10.1038/srep44027

**Published:** 2017-04-06

**Authors:** Md. Ashaduzzaman, Swapneel R. Deshpande, N. Arul Murugan, Yogendra Kumar Mishra, Anthony P. F. Turner, Ashutosh Tiwari

**Affiliations:** 1 Institute of Advanced Materials, UCS, Teknikringen 4A, Mjärdevi Science Park, SE-58330 Linköping, Sweden; 2 Biosensors and Bioelectronics Centre, Department of Physics, Chemistry and Biology (IFM), Linköping University, 581 83 Linköping, Sweden; 3 Virtual Laboratory for Molecular Probes, Division of Theoretical Chemistry and Biology, School of Biotechnology, Royal Institute of Technology, S-106 91 Stockholm, Sweden; 4 Functional Nanomaterials, Institute for Materials Science, Kiel University, Kaiserstr. 2, D-24143, Kiel, Germany; 5 Vinoba Bhave Research Institute, Binda-Dhokri Road, Saidabad, Allahabad 221508, India

## Abstract

Regeneration of immunosensors is a longstanding challenge. We have developed a re-usable troponin-T (TnT) immunoassay based on localised surface plasmon resonance (LSPR) at gold nanorods (GNR). Thermosensitive poly(*N*-isopropylacrylamide) (PNIPAAM) was functionalised with anti-TnT to control the affinity interaction with TnT. The LSPR was extremely sensitive to the dielectric constant of the surrounding medium as modulated by antigen binding after 20 min incubation at 37 °C. Computational modelling incorporating molecular docking, molecular dynamics and free energy calculations was used to elucidate the interactions between the various subsystems namely, IgG-antibody (c.f., anti-TnT), PNIPAAM and/or TnT. This study demonstrates a remarkable temperature dependent immuno-interaction due to changes in the PNIPAAM secondary structures, i.e., globular and coil, at above or below the lower critical solution temperature (LCST). A series of concentrations of TnT were measured by correlating the *λ*_*LSPR*_ shift with relative changes in extinction intensity at the distinct plasmonic maximum (i.e., 832 nm). The magnitude of the red shift in *λ*_*LSPR*_ was nearly linear with increasing concentration of TnT, over the range 7.6 × 10^−15^ to 9.1 × 10^−4^ g/mL. The LSPR based nano-immunoassay could be simply regenerated by switching the polymer conformation and creating a gradient of microenvironments between the two states with a modest change in temperature.

Troponin-T (TnT) is a highly sensitive and specific biomarker for myocardial injury^1^, and early detection of TnT can decrease the danger of death from heart attack. A TnT-immunosensor with a rapid response and low detection limit would be highly beneficial^2^. There would be added benefit in having a reversible immunosensor, and various approaches to achieve this have been described^3^,^4^,^5^,^6^,^7^,^8^,^9^,^10^,^11^,^12^. However, these biosensors suffered from perturbations resulting from the use of pH or ionic shifts and co-existing molecules in biological fluids. The ability to readily and reversibly control target-analyte interaction would expand possibilities not only for reuse, but also to compensate for background fluctuations.

A new generation of regenerable immunosensors^10^,^11^,^12^ having non-covalent supramolecular interactions with biomolecules has recently emerged. Such immunosensors depend on the conformational changes of temperature-responsive PNIPAAM. External physical stimuli, such as temperature, pH, ionic strength, electrical potential, etc. can readily alter the association and dissociation of analytes to regenerate immunosensors, by directly affecting properties such as solubility, swelling behaviour, redox (reduction-oxidation) states and crystalline /amorphous transitions^13^,^14^,^15^.

Gold nanorods (GNR) exhibiting LSPR phenomena are one of the most promising new materials for immunosensing due to their highly anisotropic shape, which offers superior optical properties compared to spherical nanoparticles^16^,^17^,^18^,^19^,^20^,^21^,^22^. As a result, GNRs have been a recent focus for applications based on wavelength shifts due to changes in dielectric properties in the vicinity of the modified nanorods^23^,^24^,^25^,^26^,^27^,^28^,^29^,^30^,^31^,^32^,^33^. Recently, Fernandez *et al*.^34^ have reported a regenerable plasmonic biosensor for the detection of anti-immunoglobulin G using gold nanolines, where an alkaline solution (0.3 M NaOH) was used to investigate the reversible interaction between antigen and antibody. Joshi *et al*.^35^, have also reported a regenerable biosensor based on gold nanoprisms for the detection of microRNA, but it required a long incubation time (2 h) for dehybridisation of the mircoRNA. In addition, Fernández-López *et al*.^11^ have fabricated PNIPAAM-GNR mircogel hybrids and shown reversible plasmon coupling with swollen and collapsed states of PNIPAAM. Very recently, we published an article^12^ describing the detection of TnT using a gold electrode PNIPAAM-based reversible immunosensor, based on impedance measurement, that yielded a detection limit of 0.5 ng/mL. In this case, the electrode was regenerated *in-situ* with a modest change of solution temperature between 25 to 50 °C.

Considering the change in colligative properties in solution during reversible association and dissociation of the target analyte, we aimed to fabricate a new LSPR-based reagent-less, temperature-modulated, regenerable GNR immunoassay, where a covalently bonded anti-TnT and amine functionalised GNR was treated with amine end-caped thermo-responsive PNIPAAM. Here we report on an integrated triggered nano-architecture of GNR decorated with monoclonal anti-TnT, which exhibited on/off-switching textural ability to create a highly sensitive reversible immuno interaction between 25 and 37 °C. The mechanism of the reversible association and dissociation of TnT to and from anti-TnT was studied using computational modelling based on a generalised Born free energy calculations. This model nanotechnological strategy could be very helpful in facilitating inherent transducer-enabled, regenerable immunosensing upon triggering.

## Results and Discussion

### Synthesis of anti-TnT-GNR and PNIPAAM-anti-TnT-GNR

**Figure 1:**
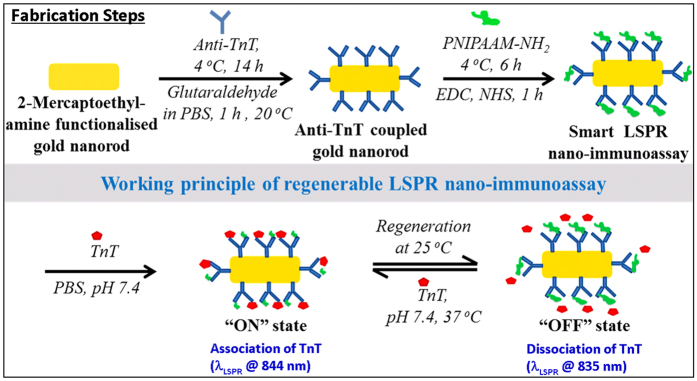
Schematic presentation of fabrication steps involved with regenerable LSPR nano-immunoassay and its reversible working mode of action at 25 and 37 °C.

Anti-TnT and amine terminated GNR were conjugated via a coupling reaction between -CHO end groups of glutaraldehyde and the respective -NH_2_ groups of anti-TnT and GNR at 4 °C. The resulting covalently bonded modified nanorod was denoted as anti-TnT-GNR. For introducing the thermal-directing behaviour into anti-TnT modified GNR, additionally amine terminated PNIPAAM was used to facilitate EDC/NHS coupling reaction^11^. The resultant PNIPAAM-modified anti-TnT-GNR structure was represented as PNIPAAM-anti-TnT-GNR. Figure 1 shows the fabrication steps and reversible conformational change of the regenerable LSPR nano-immunoassay and switchable interactions of TnT on the PNIPAAM-anti-TnT functionalised GNR surface. To verify the temperature sensitive character of TnT immunoassay, optical measurements were carried out at two different temperatures, 37 (“ON” state) and 25 °C (“OFF” state).

### Investigation of triggered nano-surface properties

Surface properties of unmodified and modified gold nanorods (GNRs) were investigated by UV-NIR spectroscopy. In the case of GNR dispersed MES buffer solution, a left peak 500 nm due to transverse, and a right LSPR peak 812 nm, due to the longitudinal mode of electron density oscillations resonance were observed, respectively^36^. These extinction peaks are mainly due to the excitation of localised surface plasmon resonances (LSPRs) in the GNRs at wavelengths corresponding to transverse and longitudinal modes of electron density oscillations. Here, anti-TnT conjugation modifies the dielectric surrounding of GNRs, which in turn changes the refractive index of a colloidal medium. A gradual decrease in *λ*_*LSPR*_ maximum with a bathochromic shift was observed as unmodified GNRs were successively modified with anti-TnT and PNIPAAM. Excitation of light for a single mode LSPR due to the induced light in the GNRs is given by the Eq. (1)^37^.


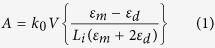


**Figure 2:**
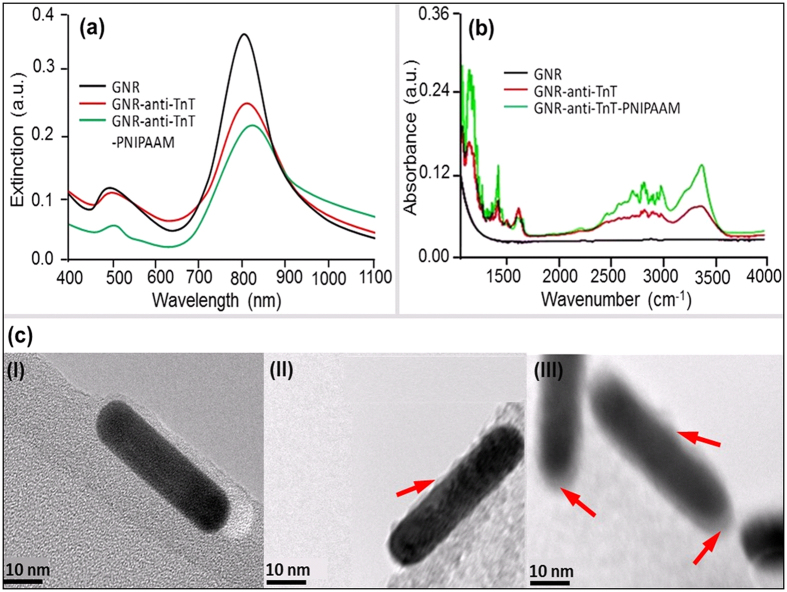
(**a**,**b**) UV-NIR, FTIR spectra and (**c**) TEM images of (I) GNR, (II) anti-TnT-GNR, and (III) PNIPAAM-anti-TnT-GNR respectively.

Where, *k*_0_ = 2π*n/λ, n* is the refractive index of the medium, *λ* is the wavelength of light*, ε*_*m*_ is the permittivity of the surrounding medium, *ε*_*d*_ is the permittivity of the nanorod, *V* is the volume of the nanorod and *L*_*i*_ is the geometrical factor of the i^th^ resonance. It is apparent from Eq. (1) that the excitation strongly depends on the shape, size and the dielectric properties of the nanorods determined along with the surrounding medium against the frequency of the incident laser. The incident light of a specific frequency induces a huge localised electric field inside the nanorods, when a frequency of the light is near to the frequency of the oscillation of an electron cloud. These electrons dissipate their energy via collisions among the particles^38^,^39^. As the GNRs are surrounded by the anti-TnT or/and polymer of a certain material index, effective dielectric properties of the nanorods are expected to shift *λ*_*LSPR*_ maxima. Because of the weak intensity, a minimal change in the resonance wavelength of the longitudinal mode is anticipated. A red shift by the modified GNRs conjugates was observed corresponding to the maxima (*λ*_*LSPR*_ at 808 nm) of unmodified GNRs. The *λ*_*LSPR*_ peaks at 817 nm (i.e., a peak shift of 9 nm) and at 831 nm (i.e., a peak shift of 23 nm) were recorded for anti-TnT-GNR and PNIPAAM-anti-TnT-GNR respectively, as shown in Fig. 2(a). These phenomena demonstrate that the NIR spectra of both modified GNRs showed a *λ*_*LSPR*_ shift to longer wavelength and exhibited the maximum shifting for GNR-anti-TnT-PNIPAAM conjugate^11^. This is most likely due to the flagging of the bonds or charges and partial ionisation over the GNR surface, which is consistent with an optical polarisation of ionic impulse and temperature responsive behaviour of PNIPAAM lids at anti-TnT. The anti-TnT based optical immunoassay works on the principle of a lock and key specific immunoreaction^35^. Thus, the optical polarisation of the ionic impulse should be encouraged by LCST of PNIPAAM for TnT sensing.

The functionalisation of GNRs, GNR-anti-TnT and GNR*-*anti-TnT-PNIPAAM was confirmed by Fourier transform infra-red (FTIR) spectroscopy, TEM, and imaging microscopy. Amide bonds formed in the functionalised GNRs were shown in two distinct sections in FTIR spectra as shown in Fig. 2(b). The amide I and II showed bands at the range: (1) 3000–3500 cm^−1^ due to stretching vibrations of N-H and: (2) 1700–1300 cm^−1^ i.e., as amide-a (between 1600 and 1700 cm^−1^ due to C=O stretching vibration), amide-b (1600–1500 cm^−1^ due to N-H bending vibration) and amide-c due to several complexes’ synchronised displacements such as the conformation of side chains and hydrogen bonding, respectively. Similarly, TEM images of three stages are shown in Fig. 2c. For example, an image of dark black smooth surface of bare GNR was taken as reference Fig. 2(c-I), whereas, a dark black core with light pulp (indicated by an arrow) of anti-TnT as a shell was found in Fig. 2(c-II). Figure 2(c-III) is demonstrated the final stage of immunosensor having GNR core structure lost darkness due to the coverage of PNIPAAM and is shown an obscure core-soft shell (indicated by the arrows) morphology. Conjugated anti-TnT changed the surface properties of GNRs which can be seen lighter GNRs images. It is noteworthy to mention that in the buffer solutions GNRs may carry certain charges which mainly play crucial role to stop irreversible aggregation. The high sensitivity of the longitudinal LSPR mode of modified GNRs with respect to their dielectric surroundings is important for the detection of TnT with respect to cardiac diseases.

### Performance study of regenerable nano-immunoassay

The recent developments in the regenerable immunosensor research offer an opportunity to improve the performance and applicability of up-to-date state-of-the-art to design the new smart bio-devices. Different approaches to regeneration of immunosensors are listed in Table 1. Most of the regenerations reported in the literature were realised by changing pH or ionic strength of system, while there are very few reports of regeneration based on the temperature regulated structural change of temperature responsive polymers, i.e., PNIPAAM. In spite of intense activities in triggered immune-regeneration study, there has been no report of an on/off-switchable LSPR transduced nano-immunoassay to manipulate antigen and antibody interactions, despite of the fact that this affords a fundamental tool if GNR is to be used in conformal transducer. Inspired by this, we functionalised GNR to make a switchable nano-immunoassay for optical recognition of TnT. To our best knowledge this is the first such report and could overlay the method for a range of other important applications.

**Figure 3:**
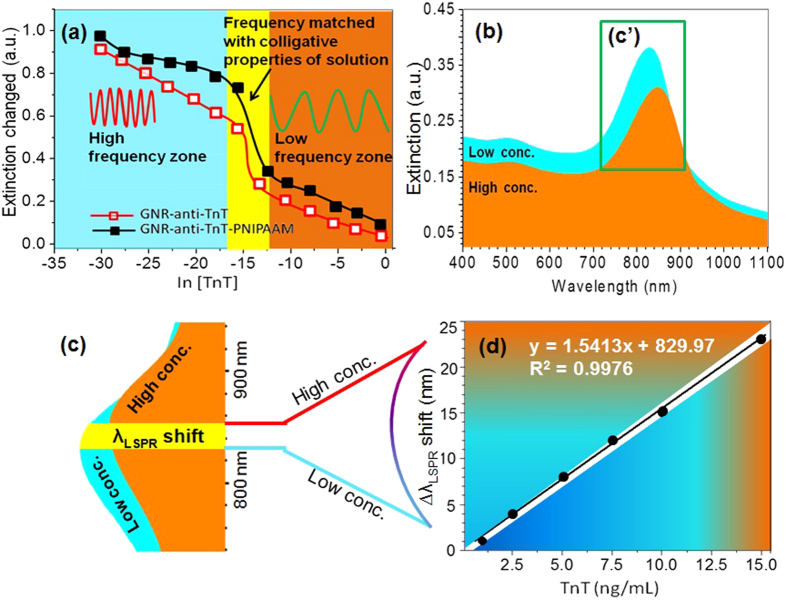
(**a**) Extinction intensity change of GNR-anti-TnT (opened square) and GNR-anti-TnT-PNIPAAM (filled square) vs. anti-logarithm of TnT concentration, (**b**) UV-NIR spectra of GNR-anti-TnT-PNIPAAM LSPR nano-immunoassay when 1 (blue) and 15 (red) ng/mL TnT solutions were treated at 37 °C, rectangular (**c′**) zone is expanded to produce (**c**) left and right boundaries of green zones are the *λ*_*LSPR*_ for 1 and 15 ng/mL TnT solution respectively and (**d**) *λ*_*LSPR*_ shift vs TnT concentration in the range of from 1 to 15 ng/mL at 37 °C.

In the present investigation, UV-NIR studies suggest that the intensity of the longitudinal LSPR mode is very sensitive with respect to TnT concentration and can be used as a tool for monitoring the troponin release. A change in extinction intensity versus anti-logarithm of TnT concentration is shown in Fig. 3(a) for GNR-anti-TnT and GNR-anti-TnT-PNIPAAM conjugates. Extinction intensity differences before and after an addition of TnT were observed with a gradual increase in TnT concentration. The black line with filled squares and red line with opened squares correspond to extinction intensity changes for GNR-anti-TnT-PNIPAAM and GNR-anti-TnT conjugates, respectively. Both the traces depict an almost linear decrease in *λ*_*LSPR*_ intensity change with increasing of TnT concentration from 7.6 × 10^−15^ g/mL to 9.1 × 10^−4^ g/mL^40^. The change in extinction intensity is observed due to the weakening of the charges/ionic potential over the GNR surface by binding of antigen. At higher temperature (37 °C), the PNIPAAM configuration is changed from coil to globular shape which may offer sufficient space for association of TnT on to the surface of GNRs, as a results the extinction peak remains sharp, i.e., the shape before association of TnT except broadening. It is also consistent with an optical polarisation of ionic impulse and temperature responsive behaviour of the PNIPAAM lids at anti-TnT with respect to concentration of TnT. The anti-TnT optical immunoassay with optical polarisation of the ionic impulse was enhanced at the LCST of PNIPAAM in the GNR-anti-TnT-PNIPAAM for TnT sensing at biological temperature, i.e., 37 °C. It is interesting to note that the extinction intensity change in the case of GNR-anti-TnT-PNIPAAM is higher that the GNR-anti-TnT^11^. The enhanced extinction intensity was due to a change in the effective refractive index of the medium, which is mainly caused by the PNIPAAM of GNR-anti-TnT-PNIPAAM conjugates. A sharp change (rapid fall) in the extinction at a concentration, ln[TnT] equals to around ~15 is very likely attributed to that fact that incident light frequency nearly matches to the collective plasmon oscillations, as determined by the effective dielectric constant of the medium. As mentioned earlier, anti-TnT-PNIPAAM conjugates showed a maximum extinction (longitudinal) at a wavelength (*λ*_*LSPR*_) ~831 nm. The peak in the longitudinal band broadened with increase of TnT concentration, while the transverse band only showed a decrease in extinction intensity with *λ*_*LSPR*_ shift of 1–2 nm at higher concentrations of TnT. The NIR spectra in the presence of two different representative TnT concentrations (1 and 15 ng/mL) are shown in Fig. 3(b), where *λ*_*LSPR*_ are observed at ~832 and ~854 nm respectively. The label-free LSPR based nano-immunoassay had a detection limit of 8.4 fg/mL with a response time of 10 sec. at 25 °C. The amplified rectangular area of Fig. 3(b) highlighted as 2(c′) is shown in Fig. 3(c) and the *λ*_*LSPR*_ for 1 and 15 ng/mL spectra is indicated by the left and right boundary of the green area, respectively. According to the results shown in Fig. 3(d), indicate the comparable values of peak shifting obtained with respect to the mentioned concentrations, which support the dissolution effects of TnT in a biological concentration. A red-shift in the *λ*_*LSPR*_ was noticed with the increase of the TnT concentration^35^. It is believed that higher TnT concentration changes effective material index eventually leading to a red shift in excitation peak.

**Figure 4:**
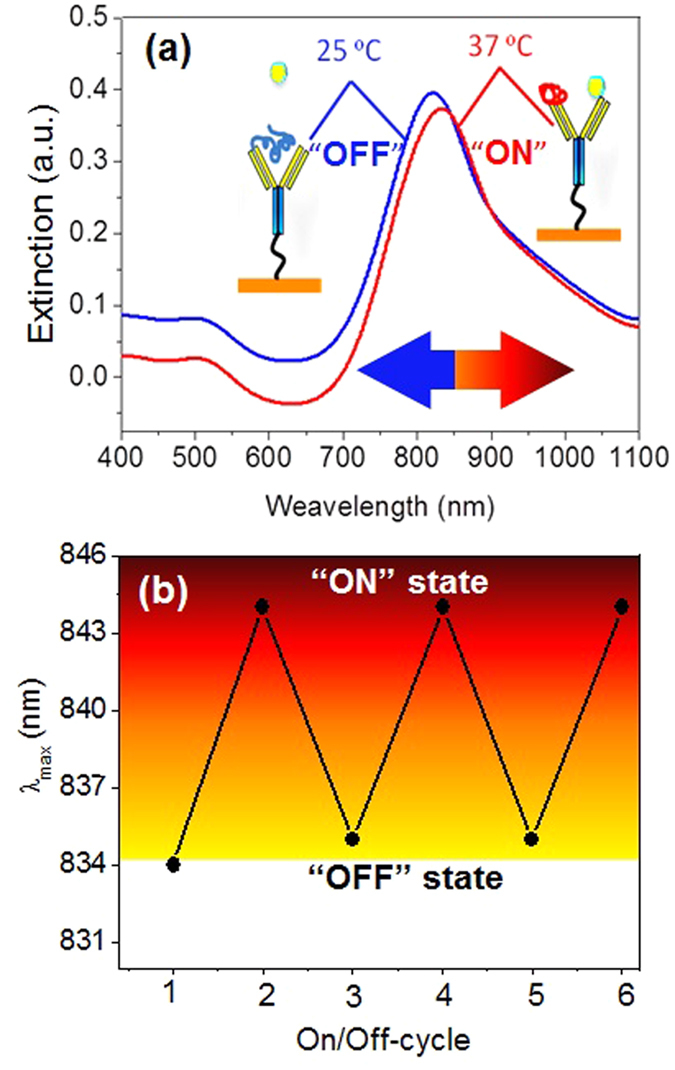
(**a**) UV-NIR spectra of GNR-anti-TnT-PNIPAAM LSPR nano-immunoassay when 5 ng/mL TnT solution was treated at 37 °C (associated) and 25 °C (dissociated). (**b**) Regeneration of GNR-anti-TnT-PNIPAAM LSPR nano-immunoassay at 37 °C and 25 °C when 5 ng/mL solution was used.

The aim of this experiment also was to observe the change in an extinction coefficient of the samples by incubating them at 37 °C for 20 min to monitor regeneration of nano-immunoassay surface by changing hydrodynamic pressure. The reversible behaviour of the GNR-anti-TnT-PNIPAAM LSPR nano-immunoassay was studied by changing the incubation temperature from 37 to 25 °C repeatedly (Fig. S1). A schematic illustration of the regenerative mechanism is shown in Fig. 4(a). At 37 °C, PNIPAAM exists in a globular form and offers available space to associate TnT, due to its reduced volume. While, at 25 °C, PNIPAAM pushes the TnT out from the anti-TnT surface. This may be due to the PNIPAAM chain in the GNR-anti-TnT-PNIPAAM conjugate that hydrates at 25 °C and hence TnT dissociates from the surface^35^.

Likewise, Fig 4(b) shows that the thermos-responsive PNIPAAM in the GNR-anti-TnT-PNIPAAM immunoassay, plays a key role in the regeneration behaviour. As expected, below the LCST of PNIPAAM (25 °C) the *λ*_*LSPR*_ for association of TnT in a 5 ng/mL solution was found at ~835 nm in all cycles except for the initial association, which occurred at *λ*_*LSPR*_ 834 nm. When the temperature was increased to 37 °C, the *λ*_*LSPR*_ peak position shifted to 844 nm periodically^35^,^41^. As the external temperature increases, i.e., above the LCST hydrogen-bonding interactions become weakened or destroyed, consequently the hydrophobic interactions of the hydrophobic moieties, i.e., –CH(CH_3_)_2_ become very strong, which induces the freeing of the entrapped water molecules from PNIPAAM and anti-TnT surface. Then TnT creates noncovalent bonding including hydrogen bonds, Van der Waals forces, ionic and hydrophobic interactions with the anti-TnT which is usually rescindable in nature. While below the LCST, PNIPAAM expands and the anti-TnT surface may be occupied via hydrogen bonding. Furthermore, the hydrophilic and ionic moieties, i.e., –CONH–, –NH^+^– and –COO^−^ may interact and co-exist through hydrogen bonding together with the antigen. It is noteworthy to mention that the free energies associated with demixing of a solution by collapsing or expanding of PNIPAAM seems to be sufficient to associate or dissociate TnT onto/from the anti-TnT surface. Palm *et al*^42^. estimated apparent binding constants from the free energy of folding (∆G) of the binary and ternary peptide complexes at 20 °C. The free energy of folding was calculated from ∆H and ∆S using the Gibbs equation, ∆G = ∆H – T∆S, in which T is the absolute temperature and ∆H and ∆S were assumed to be independent of temperature. Using the assumption that the entire difference in ∆G between the complexes and their components (∆∆G) was due to binding, the apparent dissociation constant (k_d_ = 0.43–4.91 μM) was estimated using the equation, 
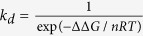
. Thus, the measurement of the strength of binding between TnT and anti-TnT is based on affinity constant (*K*a). High affinity is good for sensitive immunosensing, but too high affinity leads to irreversible sensors. Hence, when the temperature reaches or is above the LCST, the hydrophobic interactions become dominant in the GNR-anti-TnT-PNIPAAM and accordingly TnT can get access to anti-TnT at 37 °C in the immunoassay^11^. Therefore, a combination of thermo-responsive polymers together with anti-TnT is an excllent technique to construct an ultra-sensitive immunosensor with consequent regeneration of anti-TnT binding.

### Computational simulation and modelling

The reversible behaviour of immunoassay is due to the structural change of PNIPAAM which in turn modulates the interaction between the anti-TnT and Tn-T. The mechanism behind this can be understood by doing free energy calculations for various subsystems in their on and off states. Considering the relatively large time scale involved with the association process where the subsystems are polymers, anti-TnT and TnT, we have judiciously decided to investigate the free energies of individual and complexes. While it may be desirable to calculate the free energy along the coordinate for the complex (i.e., anti-TnT PNIPAAM and TnT) formation, due to the tremendous computational demands, we opted for computing the free energies of the reactants (i.e., individual subsystems) and end products (i.e., complexes), which is pragmatic approach employed in calculating the binding free energy difference of receptor-ligand complexes. The temperature- dependent structural changes in PNIPAAM were investigated using molecular dynamics with implicit and explicit solvent models. The structures for PNIPAAM obtained at temperatures at below and above the LCST were used to model its temperature specific interactions with anti-TnT and TnT. Since the regeneration behaviour of the LSPR immunoassay depends on the conformational change of PNIPAAM, we considered anti-TnT : TnT, anti-TnT : PNIPAAM and TnT : PNIPAAM complexes for free energy calculations. The role of GNRs is to serve as a sensor and as a template where different systems communicate with each other and does not contribute significantly to the energetics of these complexes and so were ignored in these calculations. Usually, the real systems are more complicated with numerous degrees of freedom and so it is common to use simplified models for calculations. Since the model fairly represents the realistic system, it is possible to get insight into a driving force for the complex formation.

To understand the temperature induced modulation of the PNIPAAM interaction with anti-TnT and TnT, it is necessary to explore the stability of various complexes formed due to these three subsystems. The free energies are computed for the individual subsystems and complexes and the binding free energy is obtained as the difference in these values. In the case of anti-TnT-TnT complex, it is defined as the Eq. (2)


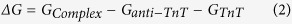


The free energy for each of these subsystems is defined as the Eq. (3).


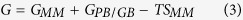


**Figure 5:**
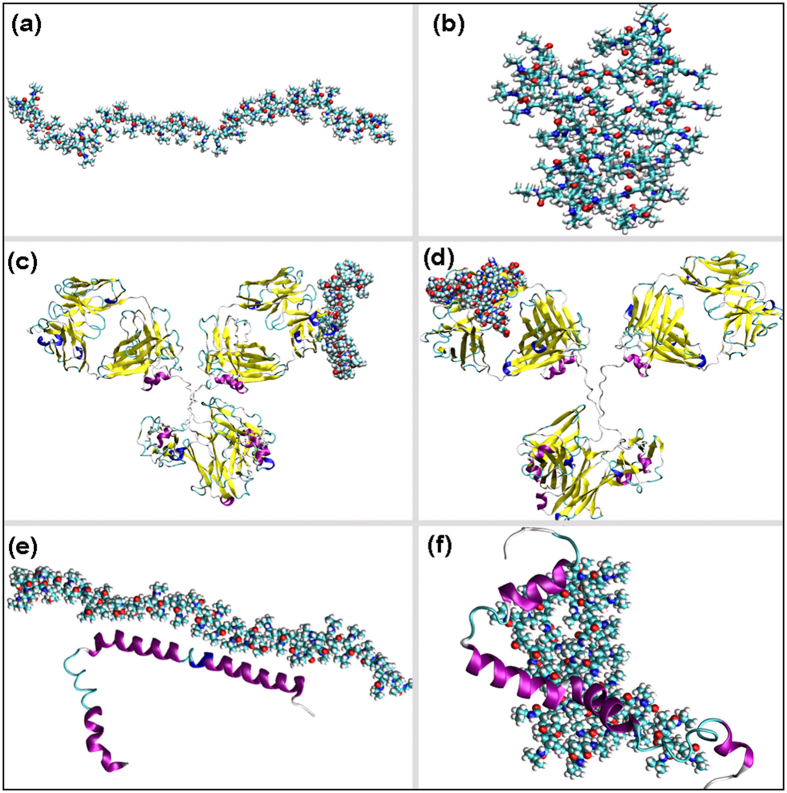
Representative figures used for free energy calculation by computational analysis; (**a**) coil-PNIPAAM, (**b**) globular-PNIPAAM, (**c**) anti-TnT-coil-PNIPAAM complex; (**d**) anti-TnT-globular-PNIPAAM complex; (**e**) TnT-coil-PNIPAAM complex and (**f**) TnT-globular-PNIPAAM complex.

Where, the first term refers to the molecular mechanics energy and includes van der Waals, electrostatic and internal energies of the system. The second term refers to the desolvation free energy that again has polar and non-polar contributions. The third term refers to the entropic contributions to the total free energy. In most of the cases, the entropic contributions do not majorly dictate the binding affinity and so the discussion is usually based on the total contributions from the first two terms. The nonpolar contribution to the desolvation free energy is computed using the solvent accessible surface area. The polar contribution is computed using either generalised Born approach (in GB approach) or Poisson Boltzmann approach (as in PB approach)^43^,^44^,^45^. Overall, these two approaches differ with respect to the polar term in the desolvation free energy. In this study, the whole discussion of free energies is based only on a generalised Born approach. We have presented the individual and total free energies (Supporting Information Tables S1–5) for five different complexes namely, anti-TnT:TnT, anti-TnT:c-PNIPAAM, anti-TnT:g-PNIPAAM, TnT:c-PNIPAAM and TnT:g-PNIPAAM as shown in Fig. 5.

Here, c-PNIPAAM and g-PNIPAAM refer to coil- and globular-like structures for PNIPAAM, respectively. Negative values of the binding free energy, which is the difference between the free energies of complex and individual systems (ΔG), show stable complex formation. The free energy for complex formation in the case of anti-TnT-TnT is −28 kcal/mol, as predicted from the MM-GBSA approach. The anti-TnT also forms a stable complex with c-PNIPAAM (the binding free energy is −14 kcal/mol), which explains the favourable anti-TnT-c-PNIPAAM association process. The g-PNIPAAM interacts with anti-TnT less strongly than c-PNIPAAM and this explains the dissociation of the complex with the coil-to-globular structural change in PNIPAAM, which is induced by temperature. It is worth noting that the interactions between anti-TnT and TnT are mostly dominated by electrostatic forces, while in the case of anti-TnT and PNIPAAM, they are mostly dominated by van der Waals interaction (refer to Tables S1 and 2 of the Supporting Information). It is clearly seen from the binding affinities of PNIPAAM with TnT (refer to Tables S4 and 5 of Supporting Information) that g-PNIPAAM interacts more strongly with TnT than the c-PNIPAAM. Moreover, the anti-TnT:g-PNIPAAM binding free energy is remarkably lower (in terms of magnitude) than that of TnT:g-PNIPAAM, which explains the preferable formation of the latter complex in the temperature range beyond LCST. However, anti-TnT-c-PNIPAAM and TnT-c-PNIPAAM binding free energies are also comparable. These results indicate that at low temperature (below LCST) PNIPAAM exists to be associated with anti-TnT. On the other hand, at high temperature (above LCST) it prefers to be associated with the TnT. Basically, the stronger TnT:g-PNIPAAM interaction makes the anti-TnT:g-PNIPAAM complex weaker followed by dissociation, which eventually leads to anti-TnT binding sites being available for TnT.

From the above interpretation, one can easily understand that the computational studies explain that the thermally-induced structural change of PNIPAAM modulates the interaction between the anti-TnT and TnT. At low temperature, the anti-TnT binding sites are not available for TnT since they are occupied by the PNIPAAM. Moreover, the binding free energies of PNIPAAM with anti-TnT and TnT are comparable and so the anti-TnT-PNIPAAM complex remains stable. However, at high temperature the structural change of PNIPAAM leads to increased interaction with TnT, which eventually leads to the breaking of anti-TnT-PNIPAAM complex and makes available free space for anti-TnT binding sites for TnT. These promising preliminary results show that a nano-immunoassay based on GNR was highly sensitive to TnT and could be used to detect this biomarker at a very early and curable stage of disease. The developed methodology is effective, inexpensive and reproducible for the detection of TnT.

## Conclusions

In conclusion, an inherent, smart LSPR nano-immunoassay has been fabricated for the detection of TnT using GNR. A linear *λ*_*LSPR*_ response to TnT concentration ranging from 7.6 × 10^−15^ g/mL to 9.1 × 10^−4^ g/mL was observed with a detection limit and response time 8.4 fg/mL and 10 sec. respectively. The surface of the anti-TnT in the immunoassay was opened and closed using thermally responsive PNIPAAM, which delivered a reversible immunosensor that could be tuned by switching the incubation temperature between 37 to 25 °C for multiple times. The free energies for complex formation in the case of anti-TnT:TnT and anti-TnT:c-PNIPAAM are −28 and −14 kcal/mol respectively, as predicted from *in-silico* modelling calculations. The c-PNIPAAM forms a stable complex with anti-TnT rather than TnT (−6 kcal/mol), which explains the mechanism of the favourable anti-TnT-c-PNIPAAM association process. Free energies of formation for different complexes within the immunosensing system clearly explain the temperature-induced structural changes of PNIPAAM that modulate the interaction between the anti-TnT and TnT to provide the differential LSPR in the GNR. This intriguing nanotechnological approach could be widely applicable in nano-LSPR based immunosensors for point-of-care devices and be relevant for other sensing and drug delivery applications.

## Materials and Methods

### Materials

Amine (2-mercaptoethylamine, 95%) functionalised, gold nanorod (GNR; > 30 μg/mL, 10 nm (DO) × 41 nm (L) ±10%, A/808 nm); monoclonal anti-troponin T (anti-TnT, IgG antibody; 3 mg/mL; rabbit skeletal muscles); troponin T (TnT, 50 μg/mL; rabbit skeletal muscles); amine terminated poly(*N*-isopropyl acrylamide) (NH_2_-PNIPAAM; *M*_n_ 2,000); glutaraldehyde (25% in H_2_O); 1-ethyl-3-(3-dimethylaminopropyl) carbodiimide hydrochloride (EDC, 99%) and *N*-hydroxysuccinimide (NHS, 99%), 4-morpholineethanesulfonic acid (MES) sodium salt, were procured from Aldrich, Schnelldorf (Germany) and used as received. The additional chemicals used were of analytical grade and solutions were prepared with Milli-Q water with a resistance of 18.2 MΩ. Amine-functionalised GNR (1.8 μg/mL) was mixed in the appropriate buffer for each conjugation reaction, vortexed for 2 min followed by ultra-sonication for 5 min to achieve a good colloidal dispersion. The GNR colloidal solution was stored at 4 °C to avoid irreversible aggregation. A stock solution of 300 μg/mL of anti-troponin and 500 ng/mL of TnT was made using PBS (0.01 M, pH 7.4) and stored at −20 °C.

### Coupling of anti-troponin onto gold nanorods (IgG-GNR)

GNR was washed in doubled distilled water followed by sonication to remove any adhered foreign materials. Then, the cleaned GNR was immersed with 100 mM of 2-mercaptoethylamine (2-MEA) in pure ethanol for 16 h at room temperature. After that it thoroughly rinsed with pure ethanol to remove the unbound 2-MEA. The GNR with self-assembled monolayer (SAM) of 2-MEA was then used for further modification steps. Monoclonal anti-TnT was covalently conjugated over the nano-surface of GNR by glutaraldehyde cross-linking reaction. A 1.8 μg/mL GNR suspension was allowed to react with 10 μL of 2.5% glutaraldehyde in PBS (10 mM) buffer for 1 h with constant stirring at 100 rpm at room temperature (20 °C). The glutaraldehyde-modified GNR was washed twice with PBS (7.4 pH) at 10,800 × g at 4 °C for 20 min. Then 150 μg/mL of anti-TnT was dropped into the solution at 4 °C and kept for 14 h. The anti-TnT conjugated nanorods were washed again twice by centrifugation at 2700 × g, for 45 min at 10 °C using PBS buffer (pH 7.4). The resultant suspension was dissolved in MES buffer and further studied for use in the nano-immunoassay.

### Fabrication of regenerable LSPR nano-immunoassay (PNIPAAM-anti-TnT-GNR)

Amine terminated PNIPAAM (50 μM) was dissolved in MES buffer by ultra-sonication followed by addition of 40 mM EDC and 10 mM NHS with continuous stirring for 1 h at room temperature. An aliquot of 150 μg of the anti-TnT conjugated gold nanorods (anti-TnT-GNR) was added to the resulting mixture, and incubated at 4 °C for 6 h. PNIPAAM conjugated antibody-gold nanorods were washed by centrifugation at 10,800 × g for 20 min at 4 °C using MES buffer.

### Characterisation

UV-NIR spectrometry was performed using a Shimadzu UV-NIR instrument using quartz cuvettes with 10 mm path length. Fourier transform infrared spectroscopy was carried out using a Vertex-FTIR spectrometer (Burker Optics, Germany) with a drop-coated calcium fluoride (CaF_2_) pellet. The morphology of nano-immunoassay was studied using a FEI Tecnai G^2^ transmission electron microscope operated at 200 kV. A copper grid holder with carbon film was immersed in the colloidal solution of samples for 45 sec and allowed to air dry at room temperature for 2 days.

### Study of LSPR nano-immunoassay

Binding inhibition of the antigen was carried out using series of TnT concentrations over the range of 7.6 × 10^−15^ to 9.1 × 10^−4^ g/mL in 1.0 M PBS at 7.4 pH. A known concentration of TnT was incubated with anti-TnT-GNR or PNIPAAM-anti-TnT-GNR at 37 °C for 20 min, respectively. Plasmon shifts and change in extinction intensities were recorded using UV-NIR spectrophotometer by scanning wavelength from 400 to 1100 cm^−1^. The nano-immunoassay was regenerated by incubation for 30 min at 25 °C.

### Computational simulation and modelling

Molecular dynamics simulations with an implicit, and then an explicit solvent model was carried out to obtain the low and high-temperature structures of PNIPAAM. For both calculations, a linear ordered structure of PNIPAAM was used as an input structure. Due to the computational cost associated with the modelling of long chain polymer, we have judiciously chosen polymer having only 60 monomer units. The structures of anti-TnT and TnT were based on the crystal structures 1IGY and 1J1E, respectively^46^,^47^. In particular, the binding site for the antibody was chosen based on existing knowledge about typical anti-TnT-TnT interaction modes^48^. However, when studying the TnT and PNIPAAM complex formation, blind molecular docking was carried out. All the docking was carried using online Hex protein-protein docking server^49^. For the most stable complex structure obtained from molecular docking, we have carried out molecular dynamics simulations in explicit solvent and further free energy calculations using the molecular mechanics/generalised Born Surface area (MM/GBSA) approach^50^,^51^. The calculations were carried out for 100 configurations obtained from molecular dynamics trajectory.

## Additional Information

**How to cite this article:** Ashaduzzaman, M. *et al*. On/off-switchable LSPR nano-immunoassay for troponin-T. *Sci. Rep.* **7**, 44027; doi: 10.1038/srep44027 (2017).

**Publisher's note:** Springer Nature remains neutral with regard to jurisdictional claims in published maps and institutional affiliations.

## Supplementary Material

Supporting Information

## Figures and Tables

**Table 1:** Reported approaches for regeneration immunosensors.

Sl. No.	Methods	Detection limit	Driving force	Ref.
1.	On/off-switchable LSPR	7.6 fg to 91 mg/mL	Temperature	**Current work**
2.	Electrochemical	0–50 mg/mL	pH	[3]
3.	LSPR	50–1000 ng/mL	pH	[4]
4.	LSPR	0.1–10 ng/mL	pH	[5]
5.	Electrochemical	1 nM	Light, pH	[6]
6.	Electrochemical, QC	0.5–5 ng/mL	Light	[7]
7.	Electrochemical, LSPR	1.1 ng/mL	pH	[8]
8.	Fluorescence	1 nM	Electro-conductivity	[9]
9.	Electrochemical, QCM, Fluorescence	2 μg/mL	Temperature	[10]
10.	Electrochemical	0.5 ng/mL	Temperature	[12]
