# Author Correction: On/off-switchable LSPR nano-immunoassay for troponin-T

**DOI:** 10.1038/s41598-022-07807-y

**Published:** 2022-03-03

**Authors:** Md. Ashaduzzaman, Swapneel R. Deshpande, N. Arul Murugan, Yogendra Kumar Mishra, Anthony P. F. Turner, Ashutosh Tiwari

**Affiliations:** 1 Institute of Advanced Materials, UCS, Teknikringen 4A, Mjärdevi Science Park, SE-58330 Linköping, Sweden; 2 Biosensors and Bioelectronics Centre, Department of Physics, Chemistry and Biology (IFM), Linköping University, 581 83 Linköping, Sweden; 3 Virtual Laboratory for Molecular Probes, Division of Theoretical Chemistry and Biology, School of Biotechnology, Royal Institute of Technology, S-106 91 Stockholm, Sweden; 4 Functional Nanomaterials, Institute for Materials Science, Kiel University, Kaiserstr. 2, D-24143 Kiel, Germany; 5 Vinoba Bhave Research Institute, Binda-Dhokri Road, Saidabad, Allahabad, 221508 India

Correction to: *Scientific Reports* 10.1038/srep44027, published online 06 April 2017

This Article contains an error. During assembly of Figure 2 during revision of the manuscript, in the panel 2c(II) the Authors introduced an overlay background which is not part of the original image. For transparency, the original image, as recorded during the experiments, is shown below as Figure 1.

**Figure 1:**
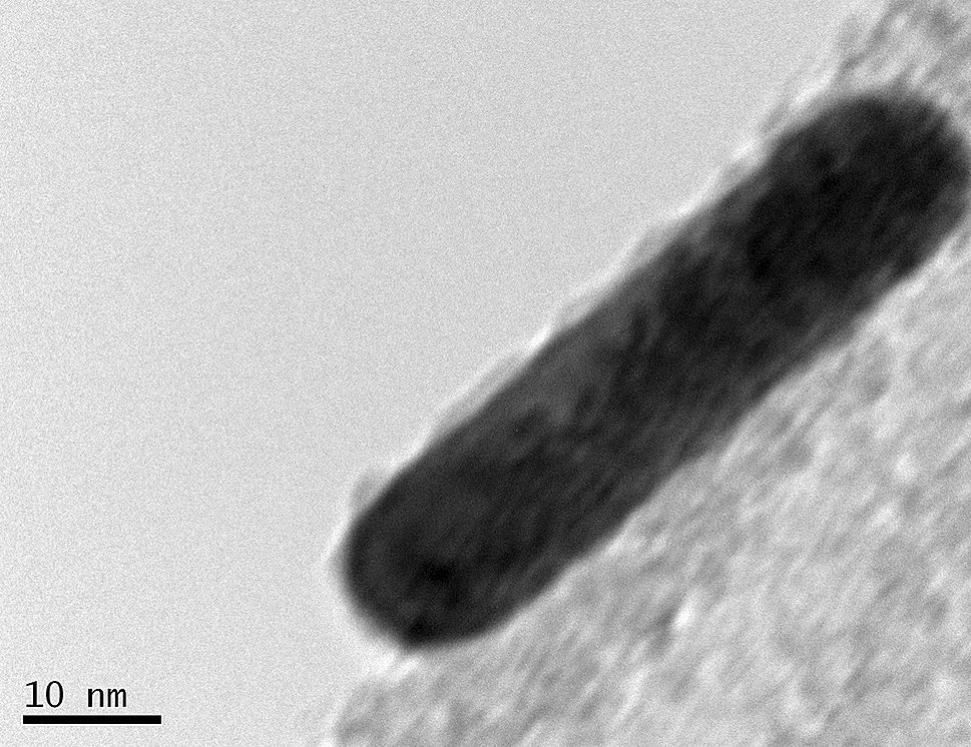
The original version of Figure 2c(ii).

This change does not affect the conclusions of the Article.

