# Supplementary material for: On/off-switchable LSPR nano-immunoassay for troponin-T
Source: Sci Rep. 2017 Apr 6;7:44027. doi: 10.1038/srep44027 (PMC5382532; doi:10.1038/srep44027)
Supplement: Supporting Information [file srep44027-s1.pdf]

Supporting Information

On/off-switchable LSPR nano-immunoassay for troponin-T

Md. Ashaduzzaman,[1,2] Swapneel R. Deshpande,[1,2] N. Arul Murugan,[3] Yogendra Kumar Mishra,[4] Anthony P. F. Turner,[2] Ashutosh Tiwari[1,2,5*]

[1]Institute of Advanced Materials, UCS, Teknikringen 4A, Mjärdevi Science Park, SE-58330 Linköping, Sweden

[2]Biosensors and Bioelectronics Centre, Department of Physics, Chemistry and Biology (IFM), Linköping University, 581 83 Linköping, Sweden

[3]Virtual Laboratory for Molecular Probes, Division of Theoretical Chemistry and Biology, School of Biotechnology, Royal Institute of Technology, S-106 91 Stockholm, Sweden

[4]Functional Nanomaterials, Institute for Materials Science, Kiel University, Kaiserstr. 2, D-24143, Kiel, Germany

[5]Vinoba Bhave Research Institute, Binda-Dhokri Road, Saidabad, Allahabad 221508, India

[*]Corresponding author.

E-mail: ashutosh.tiwari@liu.se and director@iaam.se, Tel: (+46) 13-28-2395 and Fax: (+46) 13-13-7568.

Experimental

Computational methods

Poly(*N*-isopropylacrylamide) (PNIPAAM) is known for its interesting temperature responsive property that has been utilised in various applications.[1,2] At low temperatures, it exhibits coil like structure which is stabilised due to intramolecular hydrogen bonding. At a higher temperature, it takes on a globular structure, which is stabilised due to intermolecular hydrogen-bonding with solvents.[1-3] We carried out implicit, followed by explicit solvent simulation to obtain the low and high-temperature structures of the polymer. For both calculations, a linear ordered structure of PNIPAAM was used as an input structure (refer to Figure 5a of the main article). Due to the computational cost associated with the modelling of long chain polymer we judiciously chose polymer having only 60 monomer units.

The geometry and charges for the monomer unit of PNIPAAM were obtained from density functional theory (DFT) calculations using the hybrid B3LYP functional[4] and the 6-31G* basis set[5] as implemented in the Gaussian 09 program.[6] The geometry for polymer with 60 units has been generated byrepeating units of N-isopropylacramide (NIPAAM) and bysaturating the terminal units with methyl groups. Partial atomic charges for the whole polymer unit were then fitted from the restrained electrostatic potential (RESP)[7] procedure with the constraint that the two methyl groups are neutral. The general Amber force field (GAFF)[8] was employed to model PNIPAAM.

The actual transition temperature for coil-globular structural transition depends on the system size and the rate of heating.[9] Since our aim was not to study the structural transition itself in detail, we studied the polymer at two different, well-separated temperatures. The structures at the input geometry and the representative structures for low and high-temperature PNIPAAM structures are shown in Figure 5a, 5b and 5c of the main article,

respectively. As can be seen, indeed the PNIPAAM polymer exhibits a coil-globular structural transition with increasing temperature. The final structures of the PNIPAAM corresponding to low and high temperatures were taken for the further studies on their interaction with anti-TnT and TnT. Five different molecular docking studies were carried out using Hex online server to get the structure of the most stable complex, which was then used for subsequent molecular dynamics and free energy calculations. The complex systems studied are (i) anti-TnT:TnT, anti-TnT:c-PNIPAAM, anti-TnT:g-PNIPAAM, TnT:c-PNIPAAM and TnT:g-PNIPAAM. Here, c-PNIPAAM refers to low-temperature PNIPAAM with coil-like structure. The g-PNIPAAM refers to the high-temperature PNIPAAM with globular structure.

Computational analysis

Table 1-5: The binding free energy of various complexes namely (i) Anti-TnT:TnT (ii) Anti-TnT:c-PNIPAAM (iii) Anti-TnT:g-PNIPAAM (iv) TnT:c-PNIPAAM (v) TnT:g-PNIPAAM. The various abbreviations used in the table are given in the Table footnote.

Table S1. Anti-TnT:TnT

**Energies**	**Complex**	**Anti-TnT**	**TnT**	**Difference**
Elec	-35888.2	-33252.2	-2380.5	-249.5 (3.7)
VDW	-5162.7	-4919.2	-188.7	-54.7 (0.7)
INT	29081.8	27430.6	1651.2	0.0 (0.0)
GAS	-11963.0	-10740.8	-918.0	-304.2 (3.6)
GB_{SUR}	524.8	482.5	51.1	-8.8 (0.0)
GB_{ELE}	-15775.8	-14100.8	-1960.5	285.6 (3.1)
GB_{SOL}	-15251.0	-13618.4	-1909.4	276.8 (3.1)

GB_{TOT}	-27214.0	-24359.2	-2827.4	**-27.5 (0.8)**

Table S2. Anti-TnT: c-PNIPAAM

**Energies**	**Complex**	**Anti-TnT**	**c-PNIPAAM**	**Difference**
Elec	-42925.8	-33261.3	-9655.9	-8.6 (0.4)
VDW	-4882.9	-4912.2	54.9	-25.6 (0.4)
INT	28979.0	27472.1	1506.9	0.00 (0.0)
GAS	-18829.7	-10701.3	-8094.1	-34.2 (0.5)
GB_{SUR}	535.7	480.7	58.0	-3.0 (0.0)
GB_{ELE}	-15730.1	-15373.1	-380.2	23.2 (0.4)
GB_{SOL}	-15194.4	-14892.4	-322.2	20.2 (0.4)
GB_{TOT}	-34024.1	-25593.8	-8416.3	**-14.0 (0.3)**

Table S3. Anti-TnT: g-PNIPAAM

**Energies**	**Complex**	**Anti-TnT**	**g-PNIPAAM**	**Difference**
Elec	-43265.2	-33570.2	-9688.2	-6.7 (0.6)
VDW	-5034.7	-4965.7	-30.8	-38.2 (0.6)
INT	28999.8	27493.7	1506.2	0.0 (0.0)
GAS	-19300.0	-11042.2	-8212.9	-44.9 (0.7)
GB_{SUR}	513.8	471.8	47.3	-5.3 (0.1)
GB_{ELE}	-13956.6	-13703.4	-297.4	44.4 (0.7)

GB_{SOL}	-13442.8	-13231.7	-250.1	38.9 (0.7)
GB_{TOT}	-32742.8	-24273.9	-8462.9	**-6.0 (0.5)**

Table S4. TnT: c-PNIPAAM

**Energies**	**Complex**	**TnT**	**c-PNIPAAM**	**Difference**
Elec	-12184.4	-2501.6	-9670.9	-11.9 (0.6)
VDW	-108.3	-141.2	53.8	-20.9 (0.3)
INT	3149.2	1637.7	1511.6	0.00 (0.0)
GAS	-9143.5	-1005.1	-8105.5	-32.9 (0.7)
GB_{SUR}	109.0	54.4	57.5	-2.9 (0.4)
GB_{ELE}	-2173.6	-1869.7	-333.6	29.6 (0.7)
GB_{SOL}	-2064.6	-1815.3	-276.0	26.7 (0.7)
GB_{TOT}	-11208.1	-2820.4	-8381.5	**-6.2 (0.3)**

Table S5. TnT: g-PNIPAAM.

**Energies**	**Complex**	**TnT**	**g-PNIPAAM**	**Difference**
Elec	-12182.5	-2469.3	-9677.2	-35.9 (1.0)
VDW	-229.5	-157.8	10.8	-82.5 (0.8)
INT	3110.1	1634.5	1476.4	0.0 (0.0)
GAS	-9301.9	-992.6	8190.0	-118.4 (1.2)
GB_{SUR}	89.5	49.0	51.7	-11.1 (0.1)

GB_{ELE}	-2081.7	-1850.6	-319.7	88.7 (1.1)
GB_{SOL}	-1992.2	-1801.7	-268.1	77.6 (1.1)
GB_{TOT}	-11293.2	-2794.3	-8458.0	**-40.8 (0.6)**

Elec=Electrostatic energy calculated from MM force-field, VDW=van der Waals energy, INT=Internal energy, GAS=Gas phase energy (sum of ELE, VDW, and INT), GB_{SUR} = non-polar contribution to the solvation free energy calculated by an empirical model, GB_{ELE} = the electrostatic contribution to the solvation free energy calculated by GB, GB_{SOL} = sum of non-polar and polar contributions to solvation, GBTOT = final estimated binding free energy calculated from the terms above. The values in the parentheses refer to standard error.

Anti-TnT = Anti- Troponin-T

TnT = Troponin-T

c-PNIPAAM = coil-like PNIPAAM (expanded state at low temperature)

g-PNIPAAM = globular PNIPAAM (collapsed state at high temperature)

Effect of temperature on GNR-anti-TnT-PNIPAAM

We have investigated the effect of temperature on the GNRs modified with anti-TnT and PNIPAAM in absence of TnT at pH 7.4 in MES buffer solution. It is revealed that the extinction intensity changed with temperature but there was no peak shift, this could be explained as the PNIPAAM collapsed at higher temperature and provide available bare surface of GNRs. Hence the cloud environment at the vicinity of GNR decreased, and the solution became relatively transparent as a result the only the extinction intensity slightly increased. The Fig. S1 shows the peak intensity change with temperature from 25 to 37 ^{0}C. It

is envisaged that the intensity sharply changed at 32 ^{0}C which is the lower critical solution temperature (LCST) of PNIPAAM.

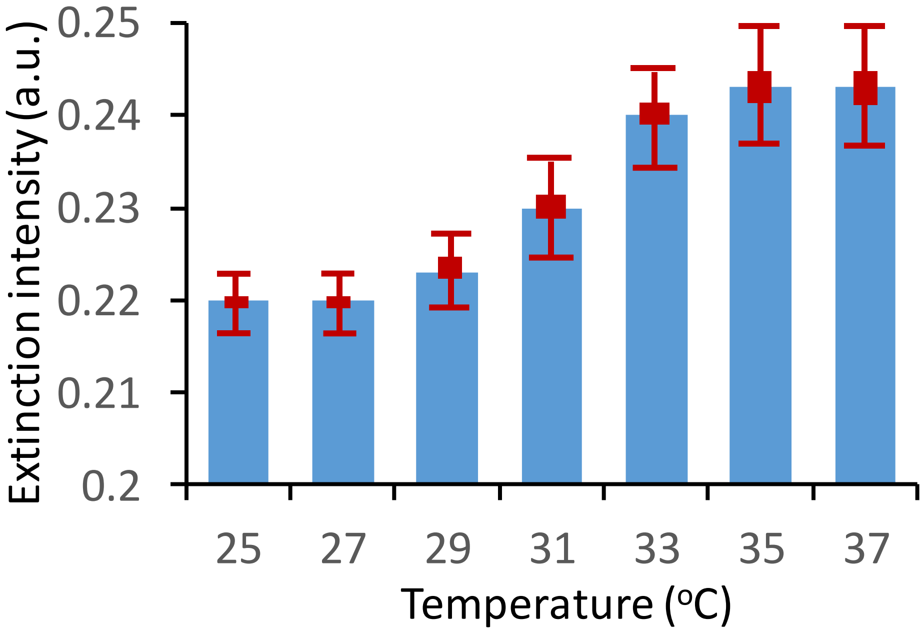

Fig. S1. Effect of temperature on GNRs-anti-TnT-PNIPAAM, when temperature was regulated at a rate of 0.2 ^{o}C/min.

References

1 H. Yim, M. S. Kent, S. Mendez, S. S. Balamurugan, S. Balamurugan, G. P. Lopez, S. Satija, *Macromolecules,* 2004, **37,** 1994-1997.

2 H.G. Schild, M. Muthukumar, D.A. Tyrrell, *Macromolecules*, 1991, **24,** 948.

3 H. Yang, R. Cheng, Z. Wang, *Polymer*, 2003, **44,** 7175.

4 A. D. Becke, *J. Chem. Phys.*, 1993, **98,** 5648−5652.

5 W. J. Hehre, R. Ditchfield, J. A. Pople, *J. Chem. Phys.*, 1972, **56,** 2257−2261.

6 Gaussian 09, Revision **D.01**, M. J. Frisch, G. W. Trucks, H. B. Schlegel, G. E. Scuseria, M. A. Robb, J. R. Cheeseman, G. Scalmani, V. Barone, B. Mennucci, G. A. Petersson, H. Nakatsuji, M. Caricato, X. Li, H. P. Hratchian, A. F. Izmaylov, J. Bloino, G. Zheng, J. L. Sonnenberg, M. Hada, M. Ehara, K. Toyota, R. Fukuda, J. Hasegawa, M. Ishida, T. Nakajima, Y. Honda, O. Kitao, H. Nakai, T. Vreven, J. A., Jr. Montgomery, J. E. Peralta, F. Ogliaro, M. Bearpark, J. J. Heyd, E. Brothers, K. N.

Kudin, V. N. Staroverov, R. Kobayashi, J. Normand, K. Raghavachari, A. Rendell, J. C. Burant, S. S. Iyengar, J. Tomasi, M. Cossi, N. Rega, J. M. Millam, M. Klene, J. E. Knox, J. B. Cross, V. Bakken, C. Adamo, J. Jaramillo, R. Gomperts, R. E. Stratmann, O. Yazyev, A. J. Austin, R. Cammi, C. Pomelli, J. W. Ochterski, R. L. Martin, K. Morokuma, V. G. Zakrzewski, G. A. Voth, P. Salvador, J. J. Dannenberg, S. Dapprich, A. D. Daniels, Ö. Farkas, J. B. Foresman, J. V. Ortiz, J. Cioslowski, Fox, D. J. Gaussian, Inc., Wallingford CT, 2009.

7 C. I. Bayly, P. Cieplak, W. Cornell, P. A. Kollman, *J. Phys. Chem.*, 1993, **97,** 10269−10280.

8 J. Wang, R. M. Wolf, J. W. Caldwell, P. A. Kollman, D. A. Case, J. *Comput. Chem.*, 2004, **25,** 1157−1174.

9 J. Wedekind, D. Reguera, R. Strey, *J. Chem. Phys.*, 2006, **125,** 214505.
